# Time-frequency analysis of band-limited EEG with BMFLC and Kalman filter for BCI applications

**DOI:** 10.1186/1743-0003-10-109

**Published:** 2013-11-25

**Authors:** Yubo Wang, Kalyana C Veluvolu, Minho Lee

**Affiliations:** 1 College of IT Engineering, Kyungpook National University, 1370 Sanyuk-dong, Daegu, 702-701, South Korea

**Keywords:** Time-Frequency analysis, Band limited multiple fourier linear combiner, Adaptive filter, Kalman filter, Smoother, EEG

## Abstract

**Background:**

Time-Frequency analysis of electroencephalogram (EEG) during different mental tasks received significant attention. As EEG is non-stationary, time-frequency analysis is essential to analyze brain states during different mental tasks. Further, the time-frequency information of EEG signal can be used as a feature for classification in brain-computer interface (BCI) applications.

**Methods:**

To accurately model the EEG, band-limited multiple Fourier linear combiner (BMFLC), a linear combination of truncated multiple Fourier series models is employed. A state-space model for BMFLC in combination with Kalman filter/smoother is developed to obtain accurate adaptive estimation. By virtue of construction, BMFLC with Kalman filter/smoother provides accurate time-frequency decomposition of the bandlimited signal.

**Results:**

The proposed method is computationally fast and is suitable for real-time BCI applications. To evaluate the proposed algorithm, a comparison with short-time Fourier transform (STFT) and continuous wavelet transform (CWT) for both synthesized and real EEG data is performed in this paper. The proposed method is applied to BCI Competition data IV for ERD detection in comparison with existing methods.

**Conclusions:**

Results show that the proposed algorithm can provide optimal time-frequency resolution as compared to STFT and CWT. For ERD detection, BMFLC-KF outperforms STFT and BMFLC-KS in real-time applicability with low computational requirement.

## Introduction

EEG uses electrodes to record electrical brain activity that originates from the post-synaptic potentials, aggregates at the cortex, and transfers through the skull to the scalp. The EEG signal is the reflection of brains neuronal oscillations. These oscillations with similar frequency and energy lead to the separation of frequency bands [1]. Numerous studies have tried to identify the relation between the frequency bands and brain states and it still remains as a hotspot of ongoing neuroscience research [2,3]. EEG activity related with voluntary movements has been the center of interest, as it is applicable to brain-computer interfaces (BCI) [4-8].

Several BCI systems rely on an amplitude attenuation phenomenon, namely event-related desynchronization (ERD) that can be voluntarily controlled by movement imagery. It was shown in [9] that during both planning and execution of hand movements, the ERD can be detected in most of the subjects within the band of *μ*-rhythm (6-14 Hz). By utilizing this amplitude attenuation phenomenon, an alternative communication pathway can be built directly from human brain to the computer [4]. The accuracy of this class of methods was examined in [6]. In recent years, this type of BCIs has been applied for limb function recovery [10] and robotic system control [7], which can improve the quality of life of the subject with severe motor function impairment. The energy decrease in ERD usually occurs in a specific frequency band for a subject. When the frequency characteristics of signal are required, the fast Fourier transform (FFT) is often used. For BCI applications, the ERD in the EEG signal is considered as a percentage change of the signal amplitude with respect to a experiment cue [9,11-13]. Since the FFT-based methods cannot provide time-frequency information, the time-frequency representation (TFR) of EEG signal is extremely important for ERD analysis.

As the performance of EEG-based BCI systems rely on the time-domain and frequency-domain features, a variety of EEG features (such as power spectrum within a pre-defined frequency band [14] and phase lock value [15]) that reflects ongoing brain states have been attempted for designing BCI systems. In general, the feature extraction algorithm requires accumulation of sufficient number of samples for generating control commands. In [16], an EEG-based BCI for three-dimensional movement control has been implemented where the power spectrum was calculated for every 50 ms with a 16-order autoregressive(AR) model. In a recent research [17], BCI with a noninvasive functional electrical stimulation has been studied. In the on-line processing phase, the BCI aggregates 500 ms EEG signal and then FFT is applied to obtain power estimates for classification. It is clear that the response time of BCIs mainly depends on the time required by the feature extraction algorithm to store and process a sufficient number of samples. Since power spectrum is a frequency domain feature, the time-frequency representation(TFR) methods that can provide amplitude variation along time axis, can be directly applied to BCI systems. Furthermore, the accuracy of BCIs can be improved by employing a narrow subject-specific frequency band [9,18,19]. In [18], an adaptive filtering approach was employed for identifying the subject-specific frequency band to improve the classification accuracy.

The TFR methods can be categorized into two types, namely non-parametric and parametric methods. The non-parametric TFRs such as band-pass filtering, short time Fourier transform (STFT) and continuous wavelet transform (CWT) were successfully applied in time-frequency analysis of EEG [20,21]. However, all the traditional methods have pros and cons in temporal and spectral resolutions. In band-pass filtering, the temporal and spectral resolution is highly dependent on the filter type, center frequency of the filter and its order. The temporal and spectral resolution of STFT is determined by the window length. The CWT can be considered as the best TFR technique among the available methods. However, it still suffers with the tradeoff between temporal and spectral resolution as STFT. The computational requirement of CWT remains as a major barrier for real-time BCI applications. A performance comparison of all the TFR methods for EEG time-frequency analysis can be found in [22].

Recently, a new method band-limited multiple Fourier linear combiner (BMFLC) was developed for estimating band-limited signals within a pre-defined frequency band [18]. By incorporating the idea of linear time varying model with a fixed frequency band, BMFLC can provide an alternative spectral estimation method for band-limited signals. The original BMFLC adopted a truncated Fourier series as the model and estimated the Fourier coefficients by least mean squares (LMS) algorithm [23,24]. As LMS requires time to converge to the steady-state, the algorithm accuracy cannot be guaranteed for small data segments. Especially, LMS algorithm is not suitable when the main objective is to accurately track the amplitude changes of a band-limited signal. To improve the accuracy of the time-frequency decomposition and the tracking ability of the existing BMFLC for real-time applications, Kalman Filter is employed. The proposed method is designed to extract time-frequency amplitude distribution that is more suitable for BCI applications. The performance of proposed method is evaluated in comparison with STFT, CWT and the existing BMFLC-LMS method.

## Methods

This section first reviews the existing methods for time-frequency representation and later presents the proposed methods.

### Classical time-frequency methods

As the traditional fast Fourier transform (FFT) does not provide time-domain information, the intuitive way to overcome this is to isolate the signal in time domain by multiplying with a window function and compute the Fourier coefficients in that time interval, then shift the time window through the time line to capture the entire time-frequency information of the signal [25].

In [26], the time-frequency resolution of STFT is interpreted by the Heisenberg uncertainty principle. It asserts that the temporal and spectral resolutions cannot be guaranteed at the same time and the joint time-frequency resolution has a lower bound given by

(1) $$△t^{2}△\omega^{2}=\frac{1}{2}$$

It can be illustrated as a box centered at (*t*,*ω*), with the length equal to △*t* in time domain and the width equal to △*ω* in the frequency domain, to sweep the whole time-frequency domain to extract the time-frequency information. Since this product remains constant, the increase of one quantity will cause degradation in the other. This degradation (leakage effect) is also due to this constant product. Thus the information that can be extracted via STFT is actually the information within that box. If the window function has a Gaussian envelope, the STFT can achieve the lower bound defined in (1) [22]. The STFT with a Gaussian window function is commonly referred as Gabor transform.

In STFT, after fixing the length of the window function, its time-frequency resolution remains constant for the entire time-frequency domain. The continuous wavelet transform (CWT) solves this problem by adopting a dilated and translated versions of the same function namely, the mother wavelet [26,27]. The dilated version of the mother wavelet is controlled by a scalar parameter *a* in CWT, where the corresponding time-frequency resolution can be provided as $\frac{△t}{a}\times a△\omega =\frac{1}{2}$. Although the time-frequency resolution is still bounded by Heisenberg uncertainty, but the length and width are scaled by the parameter *a*. Therefore, for CWT the time-frequency resolution can be adjusted by the parameter *a* compared to a fixed time-frequency resolution in STFT.

If the mother wavelet is a complex function, then the CWT is also known as complex CWT which is widely used in EEG signal processing [12,22,28]. Similar to STFT, the joint time-frequency resolution is optimized by a mother wavelet that has Gaussian envelope [22]. Morlet wavelet is one of them and is defined as [29]:

(2) $$\psi_{\text{Morlet}}(t)=\pi^{-1/4}\cdot (e^{\;j\omega_{0}t}-e^{-\omega_{0}^{2}/2})\cdot e^{-t^{2}/2}$$

where *ω*_0_ defines the oscillation of the mother wavelet. The $e^{-\omega_{0}^{2}/2}$ term in (2) is a correction term employed to satisfy the admissibility condition. If a sufficiently large *ω*_0_ is adopted, *e.g.ω*_0_>5, then the term can be neglected. The joint time-frequency resolution can achieve its lower bound with △*t*=0.707 and △*ω*=0.707 [30]. For Morlet wavelets that have a dominant periodic component, the relationship between scale and Fourier frequency [30] can be obtained as

(3) $$f=\frac{\omega_{0}}{2\mathrm{\pi a}}$$

where *f* and *a* represent the Fourier frequency and wavelets scale respectively.

The STFT and CWT belong to the analytic time-frequency representation methods, where the time-frequency resolution has a lower bound that is determined by the Heisenberg uncertainty. To apply the CWT and STFT to a signal, an appropriate length of the signal is required, as all the samples within the window function should be used at each time-frequency decomposition. The power spectrum defined in STFT and CWT is relative to the true signal spectrum that can be obtained from FFT. For some applications where the exact measurement of amplitude or power at a specific frequency is required, the CWT and STFT are not suitable.

### Band-limited multiple Fourier linear combiner

The Fourier linear combiner (FLC) proposed in [31] works by adaptively estimating the Fourier coefficients of a known base frequency together with its harmonics with the help of the least mean squares (LMS) algorithm. In [18,23], to estimate the unknown band-limited signal, a pre-defined frequency band [ *ω*_1_-*ω*_*n*_] is considered and divided into ‘n’ finite number of divisions. Then n-FLC’s are combined to form the BMFLC to estimate bandlimited signals. The frequency resolution of BMFLC, (*i.e.*$△f=\frac{△\omega }{2\pi }$), is the frequency gap between two adjacent frequency components. The selection of frequency gap is a balance between signal characteristics and analysis requirement.

The signal model adopted by BMFLC [18,32] is given by

(4) $$y_{k}=\overset{n}{\underset{r=1}{\sum }}a_{\text{rk}}\sin(\omega_{r}k)+b_{\text{rk}}\cos(\omega_{r}k)$$

where *y*_*k*_ denotes the estimated signal at sampling instant *k*. *a*_*rk*_ and *b*_*rk*_ represents the adaptive weights corresponding to the frequency *ω*_*r*_ at time instant *k*. The frequency components **x**_*k*_ and the corresponding adaptive weights **w**_*k*_ can be written in the matrix form as:

(5) $$\begin{array}{l} x_{k}=\left\{\begin{array}{c} \;\;\;{\left[\begin{array}{cccc} \sin(\omega_{1}k) & \sin(\omega_{2}k) & \cdots & \sin(\omega_{n}k) \end{array}\right]}^{T}\\ {\;\;\;\left[\begin{array}{cccc} \cos(\omega_{1}k) & \cos(\omega_{2}k) & \cdots & \cos(\omega_{n}k) \end{array}\right]}^{T} \end{array},\;\right\} \end{array}$$

(6) $$\begin{array}{c} \;w_{k}=\left\{\begin{array}{c} {\;\left[\begin{array}{cccc} a_{1k} & a_{2k} & \cdots & a_{\text{nk}} \end{array}\right]}^{T}\\ {\;\left[\begin{array}{cccc} b_{1k} & b_{2k} & \cdots & b_{\text{nk}} \end{array}\right]}^{T} \end{array},\;\right\} \end{array}$$

By employing LMS algorithm [33], the estimation of Fourier coefficients ${\hat{w}}_{k}$ can be achieved by computing the following recursive equation:

(7) $$\begin{array}{l} \;\varepsilon_{k}=y_{k}-x_{k}^{T}{\hat{w}}_{k} \end{array}$$

(8) $$\begin{array}{l} {\hat{w}}_{k+1}={\hat{w}}_{k}+2\mu x_{k}\varepsilon_{k} \end{array}$$

where *ε*_*k*_ is the error between modeling and measurement and *μ* is adaptive gain of LMS. As LMS algorithm relies on gradient based method for error minimization, the accuracy of the algorithm can be affected by the dynamic changes in the characteristics of the signal. LMS algorithm has only a single adjustable parameter for controlling the convergence rate, namely, the adaptive gain *μ*. Selection of proper *μ* is very important for stability and convergence of the algorithm. For difference choices of frequency gap (△*f*), different values of *μ* are required to ensure stability and convergence [18]. As shown in [18], BMFLC-LMS requires around 5 seconds for the initial weights to track the amplitude changes in a 25s EEG signal. For the practical BCI systems, the length of EEG signal is generally in a few seconds after the experiment cue. To improve the performance, we employ Kalman Filter (KF).

### BMFLC with Kalman filter (BMFLC-KF) for real-time estimation

**Figure 1:**
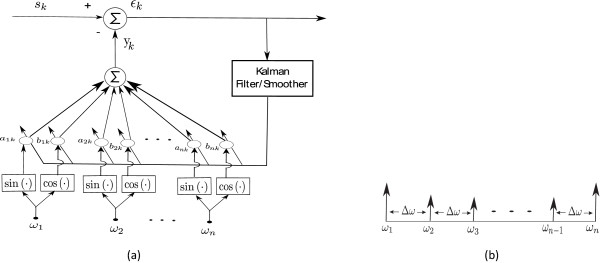
**Diagram of BMFLC. (a)** BMFLC Architecture; **(b)** Frequency components distribution.

The BMFLC signal model can be rewritten in the condensed form as

(9) $$\begin{array}{l} y_{k}=x_{k}^{T}w_{k}+v_{k} \end{array}$$

where **x**_*k*_ and **w**_*k*_ are defined in (5) and (6). Clearly, this is a linear model with **x**_*k*_ being the frequency component at each time step *k* and *v*_*k*_ the observation error. Together with (5) and (6), and the output Equation (9), we have the state-space form for the BMFLC. The architecture of the proposed algorithm is illustrated in Figure 1.

The adaptive vector **w**_*k*_ in this model is considered to be state vector. The variation of the state when no priori information is available is typically described with the random walk model [34]. The state equation can now expressed as

(10) $$\begin{array}{l} w_{k+1}=w_{k}+\eta_{k} \end{array}$$

where *η*_*k*_ is the state error.

We assume that the measurement error *v*_*k*_ and state error *η*_*k*_ are uncorrelated, zero mean, Gaussian white noise processes and are denoted as

(11) $$\begin{array}{l} v∼N(0,R) \end{array}$$

(12) $$\begin{array}{l} \eta ∼N(0,Q) \end{array}$$

where *R* and **Q** are measurement error covariance and state error covariance respectively. By employing the Kalman filter, the optimal estimation of the state of the dynamical system at time instant *k* can be obtained with the measurement sequence *Y*_1: *k*-1_=[*y*_1_,*y*_2_,⋯,*y*_*k*-1_] where *y*_*k*_ is the output defined in (4). Although the premise of the noise may not hold for the signal to be analyzed, it was shown in [35,36] that Kalman filter can also provide the minimum mean-squared error estimation within the class of linear estimators. The adaptive algorithm together with the BMFLC is shown in Figure 1.

Throughout this section, we employ the the following notation:

(13) $$\begin{array}{l} {\hat{w}}_{k}=E[\;w_{k}|y_{k-1}] \end{array}$$

where **E**[ **w**_*k*_|**y**_*k*-1_] denotes the mathematical expectation of **w** at time instant *k* with respect to previous observation **y** at *k*-1. Given the measurement sequence *Y*_1: *k*-1_, the estimated state $({\hat{w}}_{k}$ along with the estimated state error covariance **P**_*k*_) can be obtained with the Kalman filter as:

(14) $$\begin{array}{l} K_{k}=P_{k}x_{k}^{T}{\left[\;x_{k}^{T}P_{k}x_{k}+R\right]}^{-1} \end{array}$$

(15) $$\begin{array}{l} {\hat{w}}_{k+1}={\hat{w}}_{k}+K_{k}(y_{k}-x_{k}^{T}{\hat{w}}_{k}) \end{array}$$

(16) $$\begin{array}{l} P_{k+1}=[\;I-K_{k}x_{k}]P_{k}+Q \end{array}$$

with initial condition ${\hat{w}}_{0}$, **P**_0_. **K**_*k*_ is the Kalman gain updated at each time instant. The BMFLC-KF does not require the matrix inverse as it only involves a scalar observation. The proposed BMFLC-KF is computationally fast and is well suited for real-time applications.

### BMFLC with Kalman Smoother (BMFLC-KS) for off-line analysis

Furthermore, if the future measurements *Y*_*k*+1: *N*_ are available, they can be used to improve the accuracy of the state estimation. Hence the estimator is named as a smoother. In this paper, we adopt a fixed-interval smoother to improve the state estimation accuracy. The fixed-interval smoothing problem is to find the minimum mean square estimator ${\hat{w}}_{k}$ for each state **w**_*k*_(*k*=1,⋯,*N*) given the observations *y*_1_,⋯,*y*_*N*_. The smoothed estimator denoted by $w_{k}^{N}$ can be obtained as follows [35]:

(17) $$\begin{array}{l} w_{k-1}^{N}=w_{k-1}^{k-1}+J_{k-1}(w_{k}^{N}-w_{k}^{k-1}) \end{array}$$

(18) $$\begin{array}{l} P_{k-1}^{N}=P_{k-1}^{k-1}+J_{k-1}(P_{k}^{N}-P_{k}^{k-1})J_{k-1}^{T} \end{array}$$

(19) $$\begin{array}{l} J_{k-1}=P_{k-1}^{k-1}{\left[P_{k}^{k-1}\right]}^{-1} \end{array}$$

where $w_{k}^{s}=E[\;w_{k}|y_{s}]$ and $P_{k}^{s}=E[\;(w_{k}-w_{k}^{s}){\left(w_{k}-w_{k}^{s}\right)}^{T}]$ are estimated through recursion with the Kalman filter. The smoother estimation is then obtained by running the stored estimates backward in time. This procedure is suitable for off-line analysis.

The convergence properties of the proposed method are determined by the Kalman filter. The convergence analysis for autoregressive(AR) model with Kalman filter was well documented in [37-40]. It was shown in [39] that the Kalman filter is uniformly exponentially stable, if the output matrix sequence and the covariance of state error are bounded. In proposed method, the output matrix $x_{k}^{T}$ in (9) is a combination of user-defined frequency components. As sine and cosine functions are bounded, the output matrix $x_{k}^{T}$ is bounded. The covariance of state error is user-defined and is bounded. Hence the convergence of proposed algorithm can be established similar to [39]. Further the convergence rate can be quantified as in [40].

### Time-frequency decomposition with BMFLC

Based on (9) and (10), the weight vectors of BMFLC represents the Fourier coefficients of the band-limited signal. We can combine the weights into the following form:

(20) $$\begin{array}{l} w_{k}^{f}={\left[\;\;\begin{array}{ccc} \sqrt{a_{1k}^{2}+b_{1k}^{2}} & \cdots & \sqrt{a_{\text{nk}}^{2}+b_{\text{nk}}^{2}} \end{array}\right]}^{T} \end{array}$$

where $w_{k}^{\;\;f}$ is the absolute weight vector of the frequency components at time instant *k*. The absolute time-frequency weights decomposed matrix **D** can be obtained for the signal with *m* samples as

(21) $$\begin{array}{c} D\;=[w_{1}^{f}\;\cdots w_{k}^{f}\;\cdots w_{m}^{f}]\;=\;\left[\;\begin{array}{ccc} \sqrt{a_{11}^{2}+b_{11}^{2}} & \cdots & \sqrt{a_{1m}^{2}+b_{1m}^{2}}\\ \sqrt{a_{21}^{2}+b_{21}^{2}} & \cdots & \sqrt{a_{2m}^{2}+b_{2m}^{2}}\\ \vdots & \ddots & \vdots \\ \sqrt{a_{n1}^{2}+b_{n1}^{2}} & \cdots & \sqrt{a_{\text{nm}}^{2}+b_{\text{nm}}^{2}} \end{array}\right] \end{array}$$

where each row vector presents the amplitude variation of a single frequency component at each time instant *k*. The energy distribution in the time-frequency mapping can be obtained as

(22) Power=D⊙D

where the operator ⊙ represents the element by element multiplication of the matrix. The **D** matrix provide the time-domain information of all individual frequency component and it can be directly used for time-frequency representation.

Comparing with the LMS based BMFLC in [18,32], by adopting the Kalman filter combined with the smoother procedure, an accurate weights adaption process in BMFLC can be achieved. Hence it provides an accurate time-frequency decomposition. The usage of smoother is optional and it depends on the purpose of analysis. For the off-line analysis, if an accurate time-frequency mapping is required, the smoother procedure can be employed.

### Data sets

**Figure 2:**
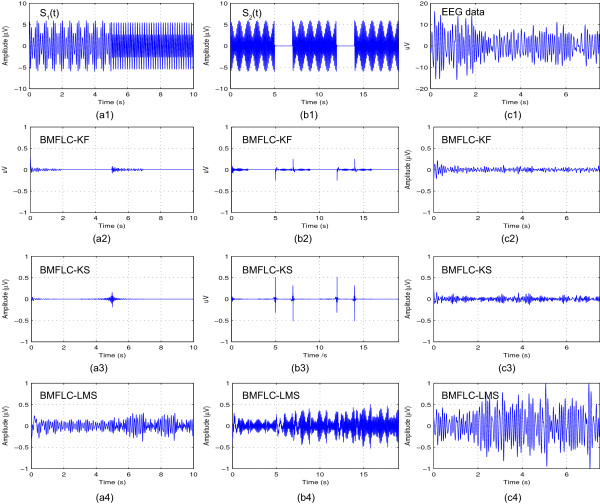
**Time-domain estimation accuracy of BMFLC, *Δf = 0.5* Hz. (a1)**, **(b1)** and **(c1)** are the synthetic signals *S*_1_(*t*), *S*_2_(*t*) and EEG respectively; **(a2)**, **(b2)** and **(c2)** are the estimated errors for BMFLC-KF; **(a3)**, **(b3)** and **(c3)** are the estimated errors for BMFLC-KS; **(a4)**, **(b4)** and **(c4)** are the estimated errors for BMFLC-LMS.

In order to compare the temporal and spectral resolutions, three synthesized signals are used in this study. Since EEG *μ*-band (6-14)Hz is of special interest, three synthesized signals are chosen to contain the frequency components within this frequency band. The first synthesized signal is defined as:

(23) $$S_{1}(t)=\left\{\begin{array}{c} \;4\text{sin}(2\pi 9t)+2\sin(2\pi 11t);\;0\leq t<5\\ \;2\text{sin}(2\pi 7t)+4\text{sin}(2\pi 14t);\;5\leq t\leq 10 \end{array}\right)$$

and is illustrated in Figure 2(a1). The signal has discontinuities in frequencies and has both amplitude and frequency modulations. This signal is used to test the frequency resolution and tracking ability of the algorithm. The second synthesized signal is defined as

(24) $$\begin{array}{c} S_{2}(t)=\left\{\begin{array}{c} 4\sin(2\pi 10t)+2\sin(2\pi 9t);\;0\leq t\leq 5,7\leq t\leq 12,14\leq t\leq 20\\ \;\;\;0;\;\;\text{otherwise} \end{array}\right) \end{array}$$

and is shown in Figure 2(b1). The sudden burst in the signal is well-suited to test the temporal resolution of the method. To further analyze the spectral resolution capabilities of all the methods, a signal with four closely spaced frequency components is chosen as

(25) $$\begin{array}{l} S_{3}(t)=4\;\text{sin}\;(2\pi 8.2t)+3\;\text{sin}\;(2\pi 8.6t)+2\;\text{sin}\;(2\pi 9t)\;\\ \;+4\;\text{sin}(2\pi 9.6t)\; \end{array}$$

For the analysis of steady-state behavior of BMFLC based methods, an extended version of signal *S*_1_ is chosen as

(26) $$S_{4}(t)=\left\{\begin{array}{c} 4\;\sin\;(2\pi 9t)+2\;\sin\;(2\pi 11t);\;0\leq t<60\\ \;2\;\sin\;(2\pi 7t)+4\;\sin\;(2\pi 14t);\;60\leq t\leq 120 \end{array}\right)$$

**Figure 3:**
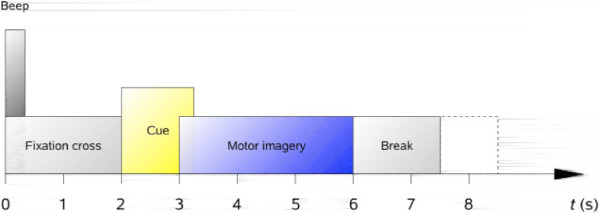
Timing scheme of the experiment.

The EEG data set used in the study is from Brain Computer Interface Competition IV [41]. The set contains EEG data of 9 subjects. EEG was recorded from 22 Ag/AgCl electrodes sampled at 250 Hz. All signals were recorded monopolarly with the left mastoid as reference and right mastoid as ground. Four classes of cue-based motor imagery tasks were carried out, namely the imagination of movement of the left hand, right hand, both feet and tongue. Each subject data was recorded in 2 sessions on separate days. Each session consists of 6 runs separated by short breaks. One runs consists of 48 trials. During the recording, the subjects sat on a comfortable armchair in front of a computer screen. At the beginning of each trial (*t*=0*s*), a fixation cross appeared on the black screen. Two seconds later, a cue in the form of an arrow pointing either to the left, right, down or up displayed on the screen and lasted 1.25*s*. The subjects were asked to perform the motor imagery task until the fixation cross disappeared from the screen at *t*=6*s*. The sequence is shown in Figure 3 (reproduced from [41]). For more details about data collection, see [41]. In this paper the hand movement imagery is considered. For the hand movement imagery, EEG data from the electrodes C3 and C4 placed over the sensorimotor cortex according to 10/20 international system, where the *μ*-rhythm originates, is selected for analysis. To limit the analysis to *μ*-band, the data was filtered between 6 Hz and 14 Hz by a fifth-order Butterworth bandpass filter.

To quantify the performance of BMFLC based algorithm, we employ the root mean square defined as $\text{RMS}(s)=\sqrt{\left(\overset{k=n}{\underset{k=1}{\sum }}{\left(s_{k}\right)}^{2}/n\right)}$ where *n* and *s* are the number of samples and input signal respectively. *s*_*k*_ denotes the input signal at time instant *k*. The percentage accuracy is defined as

(27) $$\mathrm{RMS\%}=\frac{\text{RMS}(s)-\text{RMS}(e)}{\text{RMS}(s)}\times 100$$

where *e* is the estimation error.

### Parameter selection

For estimation of synthesized signals and EEG, we set the following parameters for BMFLC based algorithms: *f*_1_=6 Hz, *ω*_1_=2*πf*_1_,*f*_*n*_=14 Hz, *ω*_*n*_=2*πf*_*n*_. Frequency spacing is set to be △*f*=0.5 Hz [18]. The weights are initialized with 0, *i.e.***w**_0_=**0**. For the BMFLC-LMS algorithm, the adaptive gain *μ*=0.035 is chosen for optimal performance for the corresponding frequency spacing △*f*=0.5 Hz [18]. The co-relation between the parameter *μ* and step-size is discussed in [18].

**Figure 4:**
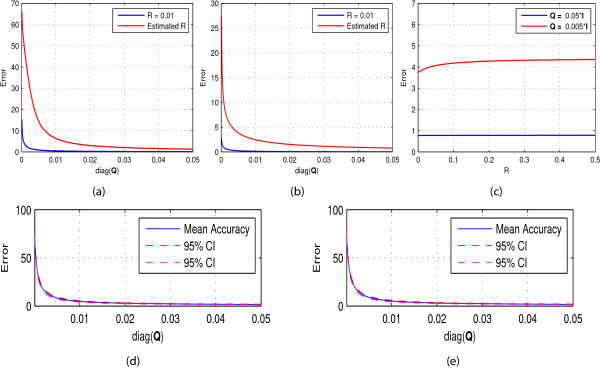
**Parameter tuning for Kalman filter. (a)** Parameter *q* selection based on *S*_1_(*t*); **(b)** Parameter *q* selection based on *S*_2_(*t*); **(c)** Parameter *R* selection with *S*_1_(*t*) for fixed *q*; **(d)** Subject #1 C3(all trials); **(e)** Subject #1 C4(all trials).

For implementation of BMFLC-KF/KS, the two parameters, the state noise covariance **Q** and measure noise covariance *R* should be properly tuned. In the following, several experiments are conducted for identification of parameters to achieve better accuracy. To start with, we assume that the state noise covariance is a diagonal matrix in the form of **Q**=*q*∗**I**. Then the parameter *q* is selected such that the root-mean-square (RMS) error is minimized. In [34], the measurement noise covariance *R* was estimated online by using the innovation process of the Kalman filter. Then an optimal value for *q* is selected to minimize the RMS error. Further, the selection of *q* is also performed for pre-fixed *R*. Experiments are first performed with synthetic signal *S*_1_ and *S*_2_ and the corresponding results are shown in Figure 4(a) and 4(b). It shows that when *q*>0, the RMS error is below 3% when *R* is estimated online and 1% for pre-fixed *R*. Based on the result of earlier experiment, we initialize *q*=0.05 and then optimize *R*. The results obtained for signal *S*_1_(*t*) are shown in Figure 4(c). As we vary the value of *R*, the RMS error remains constant. This further shows that the error performance of BMFLC-KF/KS is highly dependent on the selection of *q*.

To further justify the selection of parameters, experiments are conducted with the EEG data. EEG data of all trials of subject 1 are selected. Similar to the earlier, *R* is estimated in the algorithm, and the *q* is selected based on the RMS error. Based on the RMS error obtained for all selections of q and for all trials of the subject #1, the 95% confidence interval (CI) is estimated. To obtain reliable estimation of CI, Bootstrap method [42] with 2000 re-sampling is employed and the results are shown in Figure 4(d)-(e). Hence, we select *q*=0.01 and *R*=0.01 for optimal performance.

The STFT and CWT are set to have the same frequency resolution as BMFLC. The window function in STFT is set according to

(28) $$L=\frac{F_{s}}{△f}$$

where *L* denotes the length of the window function, *F*_*s*_ is the sampling frequency and △*f*=△*ω*/2*π*. The step size of STFT is 1/*F*_*s*_. For the wavelet-based TFR method, the Morlet wavelet is employed with *ω*_0_=6 to offer good trade-off between temporal and spectral resolutions [22]. A total of 17 scales are calculated in this paper, which are equally spaced within the range of 6*Hz* to 14*Hz* with the same frequency gap employed in BMFLC and STFT. The wavelet scale is transformed to Fourier frequency with (3).

## Results

In this section, we provide comprehensive analysis of all the five methods, BMFLC-LMS, BMFLC-KF, BMFLC-KS, STFT and CWT for synthetic and EEG data sets discussed in earlier section.

### Estimation accuracy

To compare the estimation accuracy of BMFLC based methods, the estimated signal together with the estimation error for synthesized signals (23, 24) and single trial EEG data (C3 electrode for right hand movement imagery) are shown in Figure 2.

In Figure 2(a2) and Figure 2(b2), error can be observed at the transition points for BMFLC-KF. By comparing the results of BMFLC-LMS in Figure 2(a4) and BMFLC-KF/KS in Figure 2(a2) and (a3), it is clear that the estimation accuracy depends on the adaptive algorithm. For the EEG data, BMFLC-KF/KS performed better compared to BMFLC-LMS as shown in Figure 2(c4).

For BMFLC-KS, the error is backward averaged to obtain the smoothed estimation. Since the system transition matrix is modelled as identity matrix, the accuracy of the algorithm cannot be improved with Kalman-smoother [36]. However, we can observe from Figure 2(b2) and Figure 2(b3) that the transient performance can be improved.

The RMS% error for all methods is tabulated in Table 1. The results indicate that the proposed method accurately models both the synthesized signals and EEG data. For a large EEG data set, the accuracy remained nearly constant with small variation.

**Table 1:** Estimation accuracy of BMFLC based methods

**Methods**	**Signal**					
	*S*_1_(*t*)	*S*_2_(*t*)	*S*_3_(*t*)	EEG ^*a*^	C3_RH_All ^*b*^	C4_RH_All ^*b*^
BMFLC-KF	99.47	99.39	99.49	99.22	99.19 ±0.14	99.03 ±0.20
BMFLC-KS	99.53	99.12	99.44	99.19	98.87 ±0.27	98.80 ±0.34
BMFLC-LMS	96.60	94.26	96.68	93.68	93.42 ±0.69	93.55 ±0.71

^*a*^*Single trial EEG data is considered.*

^*b*^*The 68 trials data of subject 3 right hand movement imagery is considered.*

To analyze the effect of frequency gap on estimation accuracy, the RMS% accuracy for all methods for various choices of frequency gap is computed with 68 trials of EEG data (C3_RH_All). Table 2 shows that the choice of frequency gap does not effect the accuracy of the estimation. However, the selection of frequency gap affects the frequency tracking that can be obtained from BMFLC. This issue will be discussed in the following section.

**Table 2:** Estimation accuracy for different frequency gaps

**Methods**	**Frequency gap in BMFLC**			
	△*f*=0.1 Hz	△*f*=0.2 Hz	△*f*=0.5 Hz	△*f*=1 Hz
BMFLC-KF	99.80 ±0.03	99.63 ±0.05	99.19 ±0.14	98.50 ±0.11
BMFLC-KS	99.20 ±0.10	99.03 ±0.15	98.87 ±0.27	99.07 ±0.15
BMFLC-LMS	96.93 ±0.22	95.80 ±0.40	93.42 ±0.69	96.16 ±0.77

### Temporal and spectral resolution: comparison of all five methods

**Figure 5:**
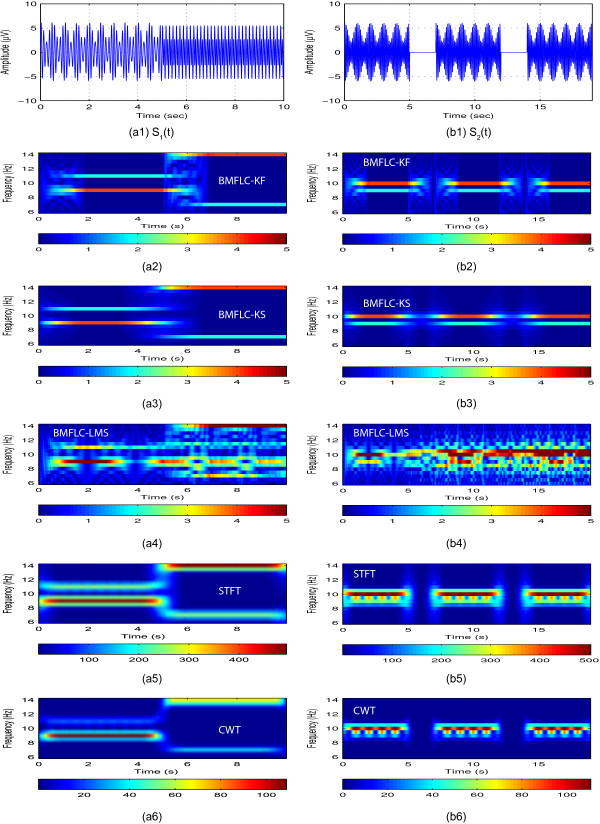
**Time-Frequency mapping for synthesized signals, *S***_***1***_***(t)* and*S***_***2***_***(t)*,*Δf=0.5* Hz.(a1)** and **(b1)** are the synthetic signal *S*_1_(*t*) and *S*_2_(*t*) respectively; **(a2)** and **(b2)** are the time-frequency mappings obtained with BMFLC-KF; **(a3)** and **(b3)** are the time-frequency mappings obtained with BMFLC-KS; **(a4)** and **(b4)** are the time-frequency mappings obtained with BMFLC-LMS; **(a5)** and **(b5)** are the time-frequency mappings obtained with STFT; **(a6)** and **(b6)** are the time-frequency mappings obtained with CWT.

The time-frequency representation of two synthesized signals together with the single trial EEG data obtained with STFT, CWT and BMFLC based methods are shown in Figure 5. The leakage effects of STFT and CWT can be clearly identified (Figure 5(a5), (b5), (a6) and (b6)) in the adjacent frequency components. The colorbar represents the absolute amplitude of corresponding frequency components in the figure for BMFLC-LMS, BMFLC-KF/KS. By construction, BMFLC models the signal into individual frequency components and hence the leakage effect that occurs in STFT and CWT can be mitigated.

BMFLC-KF provides accurate spectral estimation as shown in Figure 5(a2) and (b2). However, when there is a sudden change in the frequency, BMFLC-KF requires an adapting period for tracking the spectral changes in the signal. Although the estimation accuracy is high, there is some disturbance at the frequency transition at 5 sec as seen in Figure 5(a2) and (b2). As the amplitude weights of BMFLC-KF are initialized with zero, BMFLC-KF requires an initial adaptation period. This is mainly due to the random walk model employed for the state transition in BMFLC. By comparing Figure 5(a2) with Figure 5(a3) and Figure 5(b2) with Figure 5(b3), the estimation of smoother relays on the information provided by the Kalman filter, and operates backward to smooth the errors in the estimation. It is clear that the BMFLC-KS provides improved spectral estimation.

**Figure 6:**
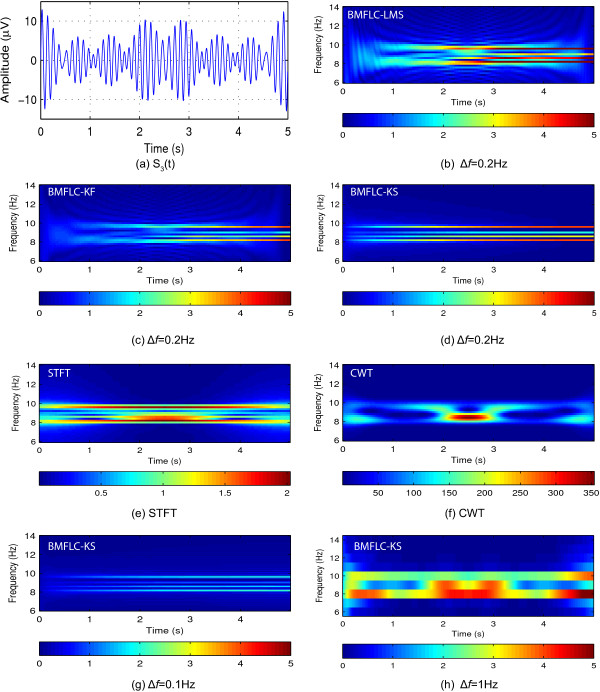
**Time-frequency mappings of various methods for synthetic signal *S***_***3***_***(t)*.(a)** Synthetic signal *S*_3_(*t*); **(b)** Time-frequency mapping of BMFLC-LMS with frequency spacing *Δf*=0.2 Hz; **(c)** Time-frequency mapping of BMFLC-KF with frequency spacing *Δf*=0.2 Hz; **(d)** Time-frequency mapping of BMFLC-KS with frequency spacing *Δf*=0.2 Hz; **(e)** Time-frequency mapping of STFT; **(f)** Time-frequency mapping of CWT; **(g)** Time-frequency mapping of BMFLC-KS with frequency spacing *Δf*=0.1 Hz; **(h)** Time-frequency mapping of BMFLC-KS with frequency spacing *Δf*=1 Hz.

Another factor that affects the spectral estimation is the selection of frequency gap *Δf*. To study the sensitivity of the method, synthesized signal *S*_3_(*t*) is employed and the results for various *Δf* are shown in Figure 6. When source signal has several frequency components located closely in the spectral domain, STFT provides better spectral estimation compared to CWT. It is also clear that, with STFT the estimated amplitude is distorted. A frequency gap of 0.2 Hz is employed for the analysis with BMFLC based methods. For a frequency gap *Δf*=0.1 Hz and 0.2 Hz, spectral estimation obtained with BMFLC-KF/KS is better compared to STFT. With BMFLC-KS the initial adaptation period is reduced and the improved performance can be seen in Figure 6(d). As the source signal *S*_3_(*t*) has closely spaced frequencies, the results for BMFLC with frequency gap 1 Hz are not accurate as shown in Figure 6(h). Furthermore, the number of frequency weights in BMFLC based methods can affect the amplitude estimation. As shown in Figure 6(g), when *Δf*=0.1 Hz gap is used, the estimated amplitude is smaller compared to the actual amplitude of the synthesized signal *S*_3_(*t*). These results clearly shows that the an appropriate frequency gap should be selected for accurate spectral estimation.

**Figure 7:**
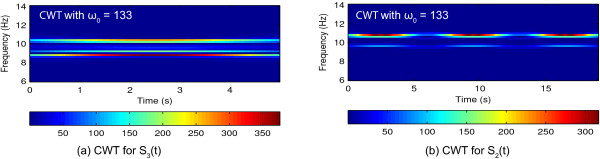
**Modified CWT for synthetic signals *S***_***2***_***(t)* and*S***_***3***_***(t)*.(a)** Time-frequency mapping of CWT for *S*_3_(*t*) with *ω*_0_=133; **(b)** Time-frequency mapping of CWT for *S*_2_(*t*) with *ω*_0_=133.

The inadequacy of CWT for the synthetic signal *S*_3_(*t*) in Figure 6(f) is mainly due to the parameter selection of the mother wavelets. The bigger the *ω*_0_ in Morlet wavelets, the better frequency resolution can be obtained. By proper tuning of *ω*_0_, an improved spectral resolution can be obtained for *S*_3_(*t*) as shown in Figure 7(a). However, for the same parameter selection, the CWT for the signal *S*_2_(*t*) is shown in Figure 7(b) and the temporal resolution is compromised. It is clear that if high accuracy is required in the spectral domain, accuracy in the temporal resolution cannot be guaranteed at the same time. Comparing the above results, BMFLC method provides better frequency resolution without loss of temporal resolution.

**Figure 8:**
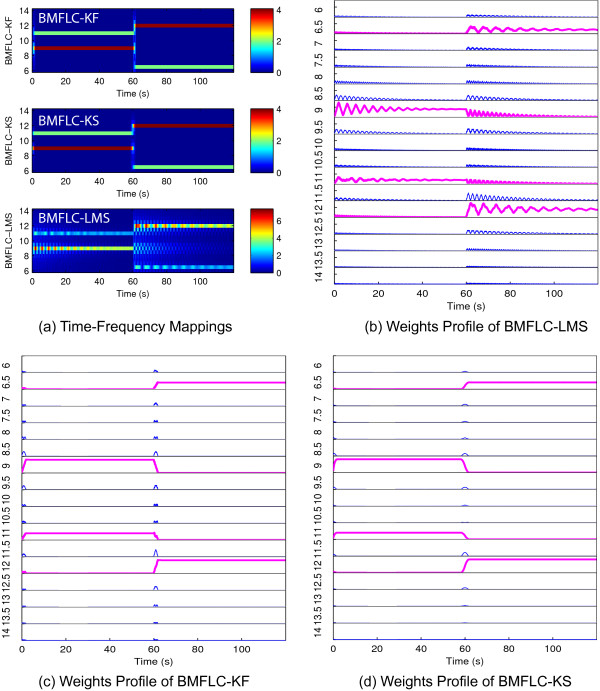
**Estimation performance for synthetic signal *S***_***4***_***(t)*.(a)** Time-frequency mappings for BMFLC-KF, BMFLC-KS and BMFLC-LMS; **(b)** Estimated weights of BMFLC-LMS; **(c)** Estimated weights of BMFLC-KF; **(d)** Estimated weights of BMFLC-KS.

Although the amplitude accuracy of BMFLC-LMS is high, its corresponding frequency components cannot adjust to the sudden changes in the frequency characteristics of the signal. The weights of BMFLC-LMS requires longer duration to track the changes in the frequency characteristics of the signal. To highlight the problem, synthesized signal *S*_4_(*t*) is employed. Time-frequency maps for BMFLC-LMS, BMFLC-KF/KS are shown in Figure 8(a). Individual weights of BMFLC are shown in Figure 8(b)-(d). It can be clearly seen in Figure 8(d) that the frequency weights of BMFLC-LMS require more time to settle to steady-state. Whereas BMFLC-KF/KS weights settle to correct frequency values immediately as shown in Figure 8(c)-(d). When the frequency components in signal *S*_4_ change at 60s, the previous settled frequency weights slowly decreases to 0 as the new frequency components gradually increase. BMFLC-KF/KS can track the sudden changes in frequency, whereas the corresponding weights in BMFLC-LMS does not settle. This clearly highlight the inadequacy of the BMFLC-LMS for extracting fast changing frequency characteristics in the signal.

### Computational complexity

In order to study the real-time applicability, the computational complexity of the TFR methods is presented. The difference between BMFLC-KF and non-parametric methods (CWT and STFT) is that they require sufficient length of input samples to be stored in order to provide the time-frequency mapping. Therefore the computational complexity of these methods relies on the length of the input data.

On the other hand, the BMFLC-KF updates the estimation at every sample. Therefore the computational complexity only relies on the observation and state-space dimensions of the Kalman filter and is independent of the input data length. The computational complexity of Kalman filter is given by *O*(3*ln*^2^), where *l* and *n* are observation and state-space dimensions respectively [43]. For STFT, the input data is first multiplied with a window function followed by FFT [44]. Hence the computational complexity of STFT is *O*(*Nlog*_2_*N*), where *N* is the length of the data being analyzed. The inverse Fourier transform for CWT requires three FFT’s for a signal with length *N*. Hence the computational complexity of CWT can be computed as *O*(3*Nlog*_2_*N*). For comparison, the operations required for all methods are tabulated in Table 3.

**Table 3:** Computational complexity

	**FFT**^***a***^	**STFT**^***a***^	**CWT**^***a***^	**BMFLC-KF**^***b***^
Notation	*O*(*N*_1_*log*_2_*N*_1_)	*O*(*Nlog*_2_*N*_1_)	*O*(3*N*_2_*log*_2_*N*_2_)	*O*(3*ln*^2^)
Operations^*c*^	10240	10240	6144	3072

^*a*^*N is the data length;N*_1_*= 1024;N*_2_*= 256;*

^*b*^*l and n are the observation and filter orders respectively.*

^*c*^*F*_*s*_*=512 Hz,* △*f=0.5 Hz.*

In BCIs, the feature required for generating a control command is extracted every 500 ms [16,45]. Let us consider the sampling frequency as *F*_*s*_=512 Hz and frequency resolution as △*f*=0.5 Hz [18]. For the given △*f*, the order of BMFLC-KF can be obtained as $n=\frac{f_{n}-f_{1}}{△f}\times 2$. In order to maintain the same frequency resolution in STFT and FFT with BMFLC, the data is padded with zeros in order to have sufficient data length (28) for analysis. For quantitative comparison, the operations required for all the methods are for a given data length is also provided in Table 3. The computational complexity of FFT [45] is also discussed in the Table. This comparison quantifies the computational complexity of the algorithms for real-time implementation. BMFLC-KF has comparatively lower computational requirement compared to other methods.

The computational complexity of BMFLC-KF depends on the dimensions of the state-space which grows linearly as the frequency gap *Δf* decreases. The computational requirement of BMFLC-KF for various *Δf* are given in Table 4. For a small frequency gap *Δf*=0.1 or 0.2, the computational complexity increases and the method becomes impractical for real-time applications. However, for EEG applications with an appropriate frequency gap, *i.e.* △*f*=0.5 Hz as in [18], the BMFLC-KF can be used for BCI applications to provide better spectral resolution with less computation as compared to STFT and CWT.

**Table 4:** Computational complexity of BMFLC-KF

**BMFLC-KF**	**△** ***f*** **=0** ***.*** **2 Hz**	**△** ***f*** **=0** ***.*** **4 Hz**	**△** ***f*** **=0** ***.*** **5 Hz**	**△** ***f*** **=1 Hz**
Operations	19200	4800	3072	768

### ERD detection

In order to test the efficiency of the proposed algorithm for BCI applications, we apply all five TFR methods to the EEG data for ERD detection. ERD detection can be identified in two ways. It can be either seen as an energy decreasing with respect to the experiment cue in the time-frequency mapping or as an energy percentage change with respect to a reference period (a pre-defined period before the experiment cue onset). The percentage value denoted as ${\text{ERD}}_{j}^{f}$ is defined similar to [42] as

(29) $$\begin{array}{l} A_{j}^{f}=\frac{1}{N-1}\overset{N}{\underset{j=1}{\sum }}{\left(w_{i,j}^{\;\;f}-{\bar{w}}_{j}^{\;\;f}\right)}^{2}; \end{array}$$

(30) $$\begin{array}{c} R^{\;f}=\frac{1}{k}\overset{r_{0}+k}{\underset{r_{0}}{\sum }}A_{j}^{f} \end{array}$$

(31) $$\begin{array}{c} {\text{ERD}}_{j}^{\;f}=\frac{A_{j}-R}{R}\times 100\% \end{array}$$

where $w_{i,j}^{\;f}$ are the estimated weights from time-frequency decomposition algorithm at *j*th sample of the *i*th trial of frequency *f*, ${\bar{w}}_{j}^{\;f}$ is the mean of weights over all trials and *N* is the number of trials/subject. *R*^*f*^ is average power in the pre-defined reference period [ *r*_0_,*r*_0_+*k*]. The reference period is generally the period before the cue is onset [9]. In line with earlier works [9,32], we select the reference period as 0 to 1.5 s before the cue onset (cue at 2 sec) for calculation of ERD. The time-frequency map for ERD is obtained by combining all the row vectors $\text{ER}D^{\;f}=\left[\;{\text{ERD}}_{1}^{\;f}{\text{ERD}}_{2}^{\;f}\cdots {\text{ERD}}_{m}^{\;f}\right]$, with *m* being the number of data samples in a given trial. The obtained ERD % is the average over all trials of a subject.

**Figure 9:**
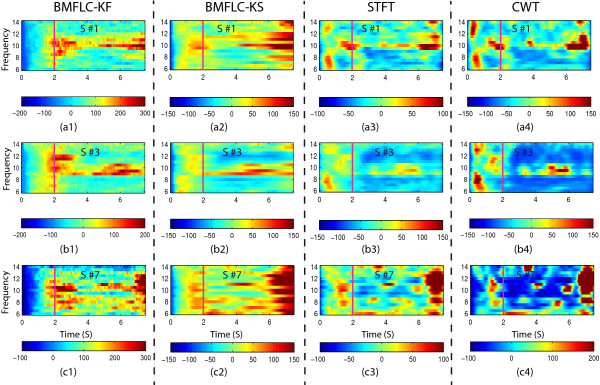
**ERD Mapping of 3 subjects for all methods. (a1)** to **(a4)** represents the ERD detection of various methods for Subject 1 (S #1) right hand imagery; **(b1)** to **(b4)** represents the ERD detection of various methods for Subject 3 (S #3) right hand imagery; **(c1)** to **(c4)** represents the ERD detection of various methods for Subject 7 (S #7) right hand imagery; Note that The magenta vertical line indicates the experiment cue onset.

The ERD detection of the corresponding electrode location and all trials for 3 subjects (Subject 1 right hand imagery, Subject 3 right hand imagery and Subject 7 right hand imagery) is shown in Figure 9. ERD pattern can be identified for all the three subjects in different frequency bands for all the methods. Comparing the results of BMFLC-KF (Figure 9(a1)-(c1)) with STFT (Figure 9(a3)-(c3)) and CWT (Figure 9(a4)-(c4)), there is a small delay in the event transition in the ERD pattern. Whereas BMFLC-KS (Figure 9(a2)-(c2)) shows similar performance compared to STFT and CWT. It is clear that BMFLC-KF requires additional 0.5s for the settlement of frequency weights (as depicted in the Figure 9(a1)-(c1) during the initial 0.5 s).

To statistically validate the performance, bootstrap with 2000 times re-sampling is used to estimate the 95% confidence interval for obtained ERD%. As stated in [42], if both confidence values of an ERD show same sign then it can be considered as significant. The bootstrap test shows that the obtained ERD mapping is significant for all methods.

**Figure 10:**
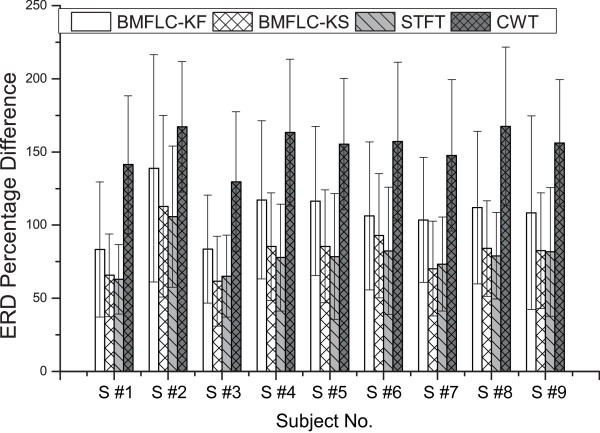
ERD detection comparison for all subjects of right hand imagery.

In order to quantify the ERD, the difference between maximal ERD percentage value and minimal ERD percentage value averaged over all frequency components is analyzed. The results are shown in Figure 10. A z-test has been applied to check whether the results are significantly different among methods. For all the subjects, the CWT shows the highest difference in ERD detection in mean value. The BMFLC-KF performs better than STFT over all the subjects (*z*=11.12,*p*<0.01) and it also outperforms BMFLC-KS (*z*=15.01,*p*<0.01). The CWT provides the best performance compared to all the four methods.

**Figure 11:**
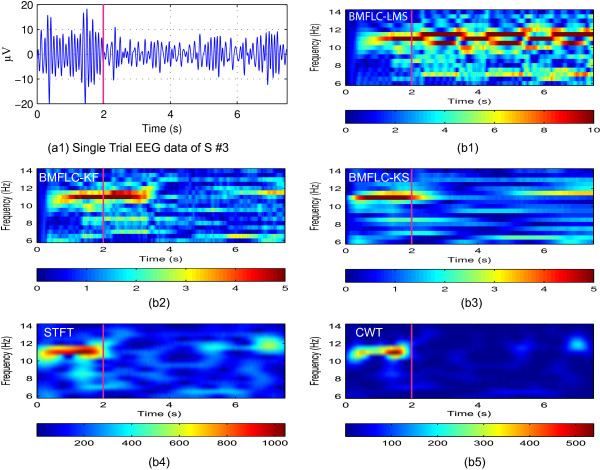
**ERD detection for Subject 3 in one trial.** The magenta vertical line indicates the experiment cue onset. **(a1)** Raw EEG signal; **(b1)** Time-frequency mapping with BMFLC-LMS; **(b2)** Time-frequency mapping with BMFLC-KF; **(b3)** Time-frequency mapping with BMFLC-KS; **(b4)** Time-frequency mapping with Short-time Fourier Transform; **(b5)** Time-frequency mapping with Continuous-wavelet Transform.

To highlight the applicability of the proposed method for BCI applications, single trial ERD pattern for subject 3 right hand imagery from C3 location (shown in Figure 11(a1)) was analyzed. ERD for a single trial data can be visually identified as an energy decreasing phenomenon after cue is onset in Figure 11 for all the methods. Since the motor sensory ERD only exists before the movement onset, this phenomenon is extremely sensitive to time delay.

By comparing the results in Figure 11(b2)-(b5), ERD can be observed in the frequency range of 10 Hz- 12 Hz in all the methods except BMFLC-LMS. Within these methods, STFT (Figure 11(b4)) and CWT (Figure 11(b5)) offer the best temporal accuracy. On the other hand, BMFLC-KF/KS also provides narrower band similar to CWT. By comparing the results of Figure 11(b1) and (b2), the adaptation period in Kalman filter can be decreased with the smoother procedure. Also, the frequency transitions in the spectral domain can be accurately estimated in the time-frequency mapping for BCI applications with the proposed method.

## Discussion

Although the proposed method ensures high accuracy for all choices of (*Δf*), an optimal selection of (*Δf*) is required for real-time implementation. For EEG, a frequency gap of 0.5 Hz is optimal [18] and ensures accurate spectral estimation. However, for off-line analysis a small (*Δf*) can be selected. The comparative study conducted on computational complexity of the TFRs shows that the proposed method has lower computational demand for a bandlimited signal. Hence, the proposed method is more suitable for accurate spectral estimation in a narrow frequency band.

While the proposed method is only discussed for a narrow frequency band in this paper, the method can still be applied for a wide frequency band. Multiple BMFLCs can be implemented in parallel by dividing the wide band into small narrow bands to ensure stability and accuracy for the algorithm. However, if the band of interests is too wide or the frequency resolution for each sub-band is too high, traditional methods would be more appropriate. A wide frequency band increases the computational requirement in BMFLC. As we confine our study to a narrow frequency band, the CWT could not provide better performance compared to STFT as the frequency resolution in CWT is scaled in the narrow band.

Heisenberg uncertainty exists in all the methods. Even though the estimation accuracy is high, the proposed method requires an additional time for the frequency weights to settle, as both amplitude and frequency cannot be estimated at the same time. The uncertainty on frequency can be clearly seen at the sudden frequency or amplitude transition. For EEG signals, an additional 0.5 s is required for the frequency weights to settle. However, it only occurs at the start of the estimation process. This initial estimation delay is inherent for all adaptive based methods and can be improved with proper initialization. The lower computational complexity of the proposed method can offset for the delay caused in the estimation compared to traditional methods. Comparatively, the CWT has the best performance in ERD detection. However, the implementation of CWT method can be a problem for real-time applications.

Although only the ERD detection is considered in this paper as application, the proposed algorithm is applicable for any bandlimited signal estimation. As ERD and ERS (event related synchronization) lies in a specific frequency bands, the proposed method can be applied for ERD and ERS detection simultaneously by employing multiple BMFLC’s in parallel. Since the weights in the BMFLC are directly related to the real amplitudes of the individual frequency components, the algorithm can utilized where an accurate amplitude estimation of a specific frequency component is required.

## Conclusions

In this research, the performance of existing BMFLC-LMS is improved by incorporating a Kalman filter. A comparison study of the BMFLC based methods with STFT and CWT is performed with both synthetic and real EEG data. The results indicate that the BMFLC-KF/KS can be used as an alternative time-frequency analysis methods for band-limited signals. As most of the frequency-based BCI applications rely on amplitude features in a fixed frequency band (*μ* rhythm) for classification, BMFLC-KF can be directly applied to most existing BCI systems. With the linear model employed, optimal estimation can be obtained with the Kalman filter. Thus the proposed method can provide an accurate time-frequency mapping with less computational complexity as compared to STFT and CWT for real-time applications. The results also show that the BMFLC-KS can provide more accurate time-frequency representation for off-line analysis.

## Competing interests

The authors declare that they have no competing interests.

## Authors’ contributions

The work was carried out as part of the Ph.D study by Mr. Yubo Wang under the guidance of Dr. Kalyana Veluvolu and Dr. Minho Lee. All authors read and approved the final manuscript.
